# The quality of metabolic pathway resources depends on initial enzymatic function assignments: a case for maize

**DOI:** 10.1186/s12918-016-0369-x

**Published:** 2016-11-29

**Authors:** Jesse R. Walsh, Mary L. Schaeffer, Peifen Zhang, Seung Y. Rhee, Julie A. Dickerson, Taner Z. Sen

**Affiliations:** 1 Bioinformatics and Computational Biology Program, Iowa State University, Ames IA, USA; 2 Electrical and Computer Engineering Department, Iowa State University, Ames IA, USA; 3 USDA-ARS Plant Genetics Research Unit and Division of Plant Sciences, University of Missouri, Columbia MO, USA; 4 Department of Plant Biology, Carnegie Institution for Science, Stanford CA, USA; 5 USDA-ARS Corn Insects and Crop Genetics Research Unit, Iowa State University, Ames IA, USA; 6 Department of Genetics, Development and Cell Biology, Iowa State University, Ames IA, USA; 7 USDA-ARS Crop Improvement and Genetics Research Unit, Albany CA, USA

**Keywords:** Metabolic pathway databases, BioCyc, CornCyc, Database comparison, MaizeCyc, JavaCycO

## Abstract

**Background:**

As metabolic pathway resources become more commonly available, researchers have unprecedented access to information about their organism of interest. Despite efforts to ensure consistency between various resources, information content and quality can vary widely. Two maize metabolic pathway resources for the B73 inbred line, CornCyc 4.0 and MaizeCyc 2.2, are based on the same gene model set and were developed using Pathway Tools software. These resources differ in their initial enzymatic function assignments and in the extent of manual curation. We present an in-depth comparison between CornCyc and MaizeCyc to demonstrate the effect of initial computational enzymatic function assignments on the quality and content of metabolic pathway resources.

**Results:**

These two resources are different in their content. MaizeCyc contains GO annotations for over 21,000 genes that CornCyc is missing. CornCyc contains on average 1.6 transcripts per gene, while MaizeCyc contains almost no alternate splicing. MaizeCyc also does not match CornCyc’s breadth in representing the metabolic domain; MaizeCyc has fewer compounds, reactions, and pathways than CornCyc. CornCyc’s computational predictions are more accurate than those in MaizeCyc when compared to experimentally determined function assignments, demonstrating the relative strength of the enzymatic function assignment pipeline used to generate CornCyc.

**Conclusions:**

Our results show that the quality of initial enzymatic function assignments primarily determines the quality of the final metabolic pathway resource. Therefore, biologists should pay close attention to the methods and information sources used to develop a metabolic pathway resource to gauge the utility of using such functional assignments to construct hypotheses for experimental studies.

**Electronic supplementary material:**

The online version of this article (doi:10.1186/s12918-016-0369-x) contains supplementary material, which is available to authorized users.

## Background

Developing a metabolic pathway resource involves many steps. These steps can be described as follows: Given a genome assembly and a gene model set, translated protein sequences are fed into a computational pipeline. Enzymes are then predicted and assigned a functional category, usually based on Gene Ontology (GO) [1] terms or Enzyme Commission (EC) [2] numbers. After the initial enzymatic function assignments are made, enzymes are then mapped to a reference metabolic pathway database to create an initial metabolic pathway resource. Finalizing a pathway resource requires manual curation to improve the accuracy of the final metabolic representation.

A wide-range of computational methods can be applied at each step of developing a metabolic pathway resource. This variance makes a comparison of metabolic pathway resources challenging. The problems that complicate comparison between heterogeneous databases have long been recognized [3], and several attempts have been made to homogenize data from different sources [4, 5]. Studies seeking to compare data content between resources [6] describe many of the challenges of matching biological data in order to assess overlap. Non-standard chemical naming conventions, difficulty matching stereo-chemistry and protonation, as well as defining pathway boundaries and managing gene variants all create challenges for comparing metabolic pathway resources.

For maize, two metabolic network resources are available, both of which are based on the B73 RefGen_v2 genome assembly/gene model set [7] and used the Pathway Tools software [8] to map enzymes onto reactions and pathways. This provides a unique opportunity to explore the effect of the initial enzymatic function assignment pipeline on the final metabolic pathway resource.

CornCyc 4.0 (http://www.plantcyc.org) was developed using the Ensemble Enzyme Prediction Pipeline (E2P2 v2.0) [9] created by Plant Metabolic Network (PMN) [10] in collaboration with MaizeGDB (http://www.maizegdb.org) [11, 12]. MaizeCyc 2.2 [13] was developed based on the Ensembl XRef pipeline [14, 15] in collaboration between two database projects, Gramene (http://www.gramene.org) and MaizeGDB. The term “Ensemble” in the CornCyc pipeline refers to integration of methods, whereas “Ensembl” in the MaizeCyc pipeline refers to the collaborative project between the European Bioinformatics Institute and the Wellcome Trust Sanger Institute.

In order to gain insight into the strengths of each resource based on initial enzymatic function assignments, we compared the data content and accuracy of CornCyc and MaizeCyc by calculating the overlap of different data types between the resources and compared the accuracy of computational annotations against experimentally-assigned enzymatic functions.

## Methods

### Gold standard protein annotation data

A gold standard set of protein functional annotations was generated by extracting data from UniProt [16] and BRENDA [17]. We extracted all protein sequence and annotation data from UniProt (release 2016_05) for the organism *Zea mays*, keeping the EC annotations only from the manually reviewed component of UniProt, while removing those annotations that had not undergone manual review. We also extracted experimentally verified protein annotations for *Zea mays* from BRENDA (release 2016.1). The UniProt and BRENDA annotations were then merged by matching proteins based on the database cross-links provided by BRENDA, resulting in the union of the reviewed annotations from UniProt and the experimentally verified annotations of BRENDA with duplicates removed. The merged protein annotations were then matched to the B73 RefGen_v2 translated gene models using BLASTP based on a sequence identity cutoff of 96% and an e-value cutoff of 1e-20. We selected the top scoring hit for each protein which resulted in matches to 1,815 unique maize proteins. EC annotations for alternate isoforms were consolidated at the gene level, resulting in 1,475 experimentally verified or manually reviewed protein functional annotations across 1,450 maize genes.

### Resource preparation and access

**Fig. 1:**
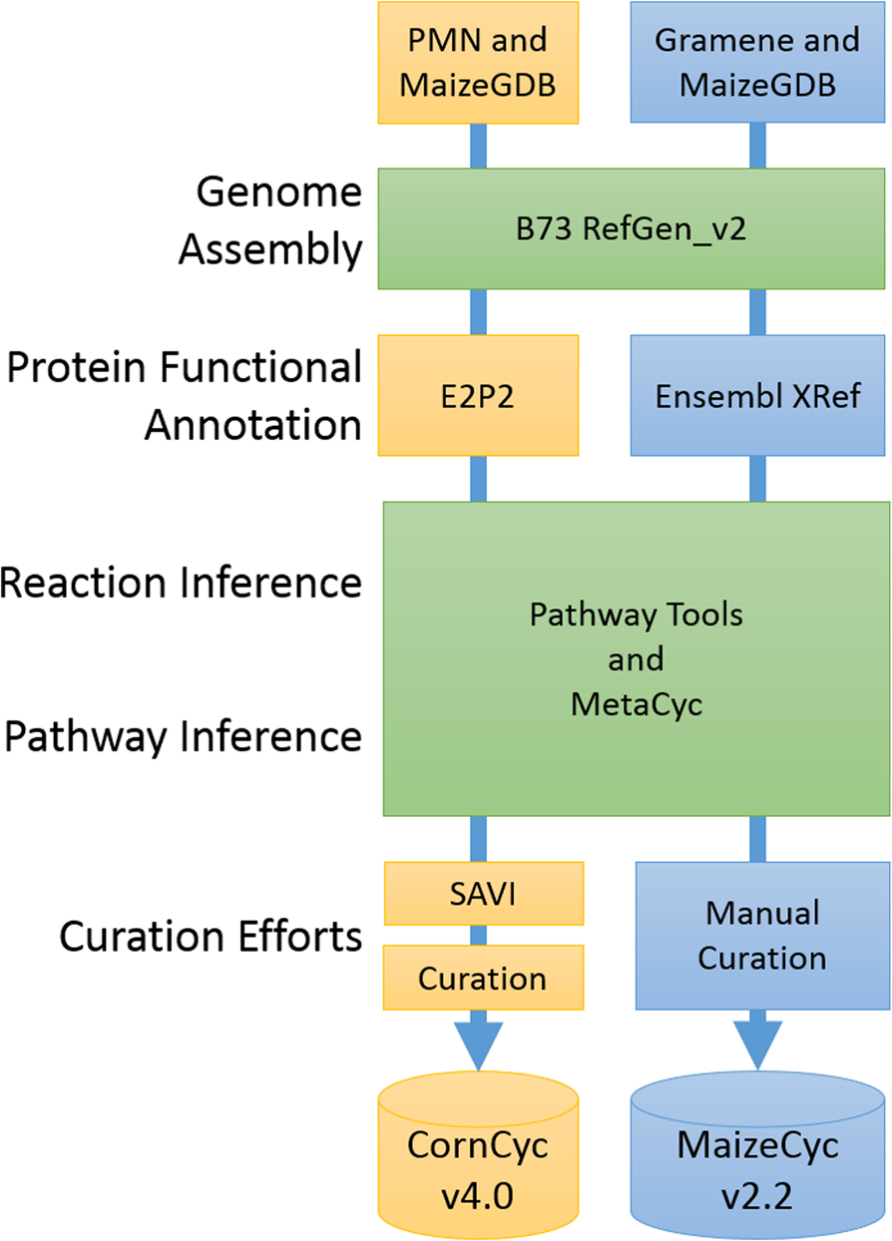
Overview of the pipelines used to create CornCyc 4.0 and MaizeCyc 2.2. *Green* represents common components, and *orange* and *blue* CornCyc- and MaizeCyc-specific components respectively. CornCyc and MaizeCyc were both based on the B73 RefGen_v2 gene model. They mainly differed in different functional annotation prediction methods incorporated into their respective pipelines. Both databases used Pathway Tools and MetaCyc for their reaction and pathway inference. Since both databases were created at different times, they used different versions of MetaCyc. Finally, manual curation has been applied to both databases. In order to account for differences at the pathway and reaction inference steps as well as at the manual curation step, we propagated updates from the same version of MetaCyc to both databases and allowed the propagation utility to remove manually curated data

We compared CornCyc version 4.0 with MaizeCyc version 2.2 hosted within Pathway Tools 17.5 [8]. Throughout the text, we refer to CornCyc version 4.0 as CornCyc and MaizeCyc version 2.2 as MaizeCyc unless otherwise specified. Figure 1 compares the pipelines used to produce both databases. Both resources are based on the B73 RefGen_v2 reference genome assembly and the filtered gene set (FGS) [7]. Although the v2 assembly of the maize genome sequence is not as recent as the v3 assembly, MaizeCyc was only available for v2, which drove our decision to use the v2 assembly and less recent CornCyc 4.0 (the current version CornCyc 7.0 uses the more recent v3 assembly). Also, while MaizeCyc was developed using Pathway Tools 15.5, CornCyc was developed using Pathway Tools 16.5. In order to make a consistent comparison, both CornCyc and MaizeCyc were upgraded to Pathway Tools 17.5 and MetaCyc 17.5 as follows: first we upgraded the schema of both CornCyc and MaizeCyc to Pathway Tools 17.5 using the built-in PathoLogic upgrade tool. Then we removed all manually curated GO terms from MaizeCyc. Finally, we used the “propagate MetaCyc updates” and “rescore pathways” procedures in order to ensure that all reactions, compounds, and pathways were up-to-date with MetaCyc version 17.5. The process of updating and rescoring pathways also served to remove existing manual curation at the pathway level, such as the application of SAVI to CornCyc. The SAVI procedure was not reapplied to either CornCyc or MaizeCyc. Removing the manually curated GO terms before the propagate and rescoring steps prevented bias that would otherwise occur. All data extraction queries to the CornCyc and MaizeCyc resources were made using the JavaCycO libraries [18] and the Pathway Tools Application Program Interface (API). Details of the methods used to extract and compare the data from CornCyc and MaizeCyc are available in Additional file 1.

### CornCyc annotation pipeline

CornCyc was developed based on the Ensemble Enzyme Prediction Pipeline (E2P2) [9]. E2P2 uses an average weighted integration algorithm based on results from individual classifiers such as BLAST [19], CatFam [20], and Priam [21]. The ensemble algorithm relies on an average weighted integration scheme where the weight of each predicted model was determined by a 5-by-3 nested cross-validation routine. For CornCyc 4.0, E2P2 version 2.1 (https://dpb.carnegiescience.edu/labs/rhee-lab/software) was used with BLAST’s e-value cutoff set to be ≤ 1e-30. The training of E2P2 and the reference databases used in the annotation process are based on the Reference Protein Sequence Dataset (RPSD) version 2.0 included in the E2P2 v2.1 package. RPSD contains protein sequences with experimental support of existence compiled from Swiss-Prot [22], MetaCyc [23], and BRENDA [17].

After the initial database generation, CornCyc was further modified by Plant Metabolic Network using the SAVI pipeline [10], which categorizes the initially predicted pathways to be retained, deleted, or manually reviewed based on a set of rules developed as a part of the curation process. SAVI also detects missing pathways. The SAVI program uses six curated pathway library files to enable semi-automated changes to a predicted pathway database (http://www.plantcyc.org/about/savi_pipeline.faces). All pathway library files used in validating and refining CornCyc 4.0 are available online at: ftp://ftp.plantcyc.org/Pathways/SAVI_validation_lists/SAVI_validation_lists_archive/SAVI_lists_pmn8_july_2013/.

### MaizeCyc annotation pipeline

The development pipeline for MaizeCyc was described in detail previously [13]. MaizeCyc is based on the B73 RefGen_v2 filtered gene set. The pipeline uses transcripts with the longest open reading frame (“the canonical transcript”) for functional annotation based on scores derived from the Ensembl XRef pipeline [14] following protein sequence alignment to UniProt [16]. Additional sources of enzymatic function annotations include classical maize genes [24], coordinates and cross-references from Maizesequence.org (now folded into Gramene), MaizeGDB (locus names/synonyms, molecular function, etc.) [11, 12], UniProtKB/Swiss-Prot [16, 22] (functional descriptions and EC assignments), Gene Ontology [1] (molecular function, biological process, and cellular location), and proteomics-supported gene annotations (e.g., cellular location). Reactions and pathways were computationally inferred using the Pathologic component of Pathway Tools [8].

## Results and discussion

### Validation of enzymatic function assignments against experiments

To determine the accuracy of the computationally predicted protein function annotations in the publicly available versions of CornCyc 4.0 and MaizeCyc 2.2, we compared the predicted annotations at the gene level against the gold standard set of annotations described in the “Methods” section. We used the following definitions for our performance classifications: 1) true positive (TP) is when a predicted function of an enzyme matches an experimentally determined function category for that enzyme. 2) False positive (FP) is when a predicted function does not match any experimentally determined function category for that enzyme. Finally 3) false negative (FN) is when a function category is an experimentally determined but is not predicted by the annotation algorithm. When counting the false negatives, we included cases when a gene is not present in CornCyc or MaizeCyc, which accounted for 86 additional false negatives in CornCyc and 2 additional false negatives in MaizeCyc. The fourth category, true negative (TN), is a quantity that is difficult to capture, as it means that for a given enzyme no prediction is made for a functional category that is also ruled out experimentally. Precision, recall, and F-measure only uses TP, FP, and FN classifications. A summary of the results is shown in Table 1.

**Table 1:** Prediction performance of CornCyc 4.0 and MaizeCyc 2.2

	True positive	False positive	False negative
CornCyc	1,326	213	149
MaizeCyc	1,235	436	240
Merged	1,365	583	62

We used the following expressions for analysis: *p* *r* *e* *c* *i* *s* *i* *o* *n*=*T* *P*/(*T* *P*+*F* *P*) and *r* *e* *c* *a* *l* *l*=*T* *P*/(*T* *P*+*F* *N*). Precision is a ratio of correctly predicted classes among all the predictions, and recall is a ratio of correctly predicted classes among all the possible correct classes. F-measure is a combination of these two measures and provides a single measure for comparing the performance of two sets of predictions. F- measure is defined as 2∗(*p* *r* *e* *c* *i* *s* *i* *o* *n*∗*r* *e* *c* *a* *l* *l*)/(*p* *r* *e* *c* *i* *s* *i* *o* *n*+*r* *e* *c* *a* *l* *l*).

**Fig. 2:**
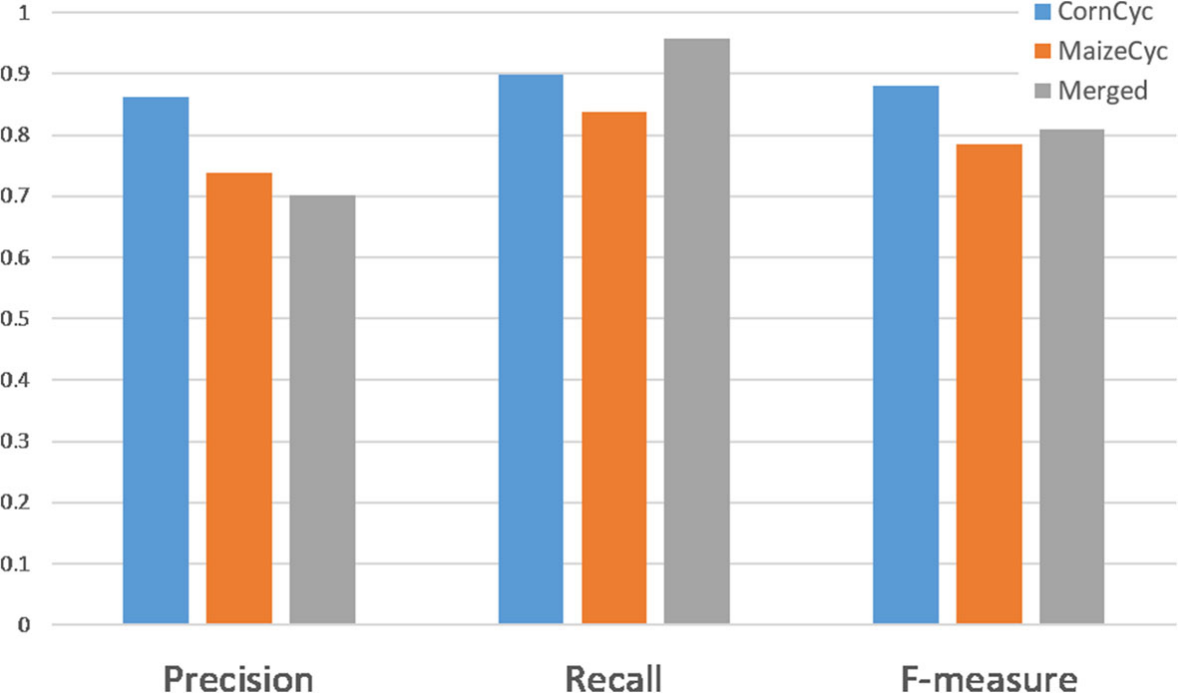
Performance comparison between CornCyc 4.0, MaizeCyc 2.2, and the union of both datasets based on 1,475 experimentally verified annotations across 1,450 genes

CornCyc performs better than MaizeCyc, as demonstrated by higher precision (0.86 versus 0.74), recall (0.90 versus 0.84), and F-measure (0.88 versus 0.79) (Fig. 2). CornCyc’s performance originates from the much higher number of true positives. For biologists, a higher F-measure means is that when they find an annotation in CornCyc, it is more likely to be correct than it is in MaizeCyc.

In order to understand how combining annotations determines the final prediction performance, we merged all the enzymatic assignments from two resources into a single resource. Figure 2 shows the performance measures for the merged resource. The merged annotations from CornCyc and MaizeCyc performed worse than CornCyc overall and better than MaizeCyc, while having greater coverage than either dataset individually.

### Comparison of data overlap

**Fig. 3:**
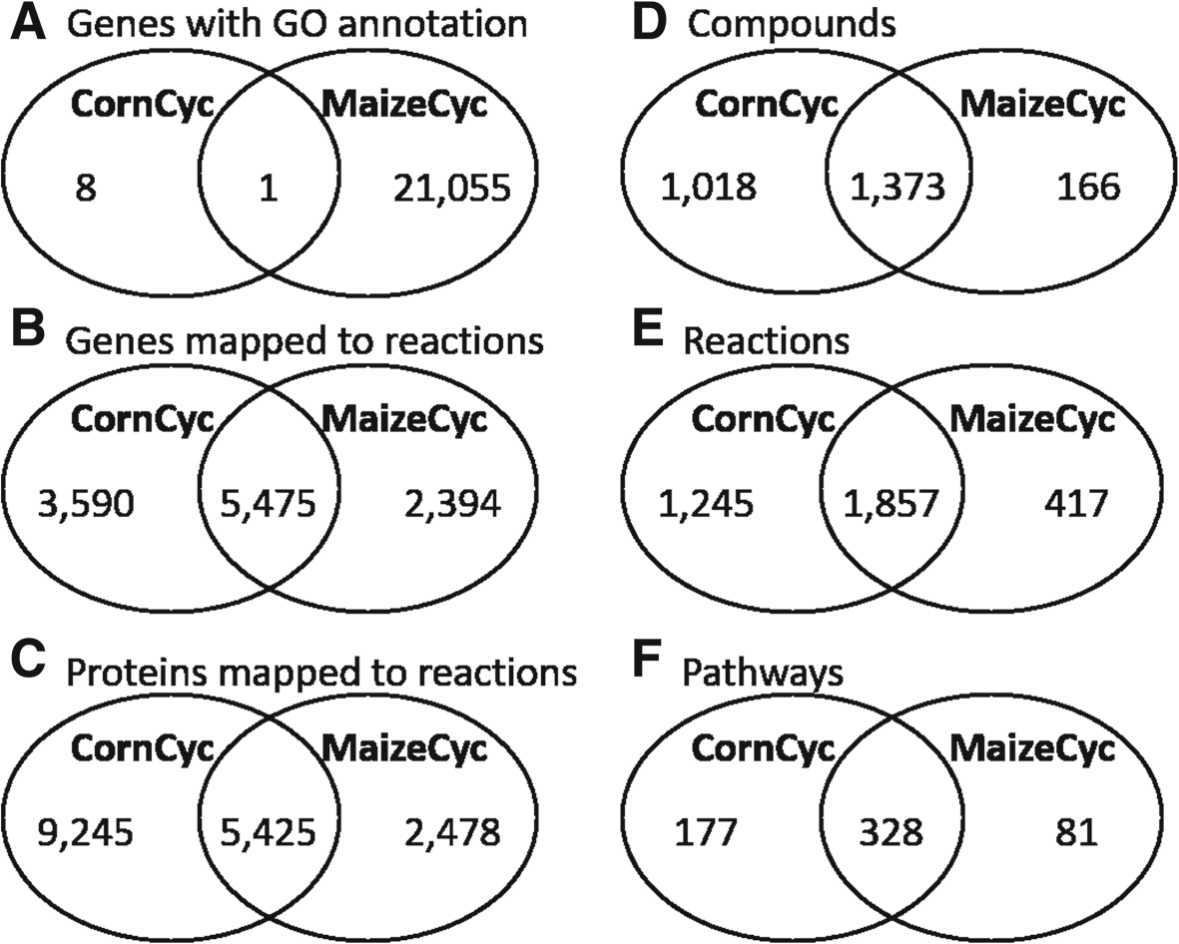
Comparison of **a**) GO annotated gene, **b**) reaction mapped gene, **c**) reaction mapped protein, **d**) compound, **e**) reaction, and **f**) pathway statistics between CornCyc 4.0 and MaizeCyc 2.2

Despite the fact that both were developed on the same gene model set, MaizeCyc and CornCyc have a quite different distribution of GO-annotated and mapped genes/proteins (Fig. 3 a–c). Part of the reason for this is that the scope of MaizeCyc includes all genes in the maize B73 RefGen_v2 filtered gene set, while the scope of CornCyc is limited to only enzyme-coding genes in the filtered gene set. In order to draw a useful comparison between the gene content in CornCyc and MaizeCyc, we only considered genes associated with a form of annotation. Specifically, we define a gene to have annotation if it is either assigned at least one GO term or is associated with a protein that catalyzes at least one reaction. In CornCyc, only 9 of the 9,142 genes have GO term annotations, but 99.1% are mapped to at least one reaction. In MaizeCyc, 53.1% of the 39,654 genes have GO term annotations while 19.8% are mapped to at least one reaction (Fig. 3 b).

MaizeCyc contains more genes/proteins than CornCyc, many of which only have GO annotations and are not associated with a reaction. CornCyc contains 1.5 times as many unique genes as MaizeCyc (Fig. 3 b), and nearly four times as many unique reaction-mapped proteins (Fig. 3 c). The difference in the number of proteins can be explained by the fact that this version of CornCyc contains, on average, 1.6 alternative splice variants per gene. In contrast, MaizeCyc includes very few splice variants.

**Fig. 4:**
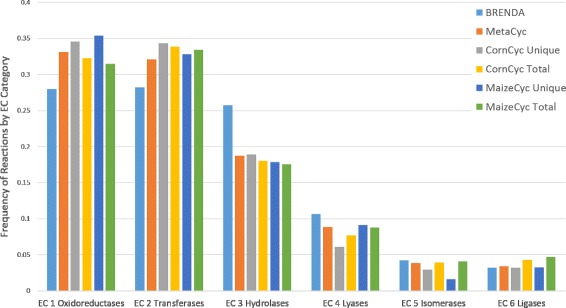
Comparison of Reactions Sorted by EC Category between CornCyc 4.0, MaizeCyc 2.2, BRENDA (July 2015 Release), and MetaCyc 19.5. For CornCyc and MaizeCyc, reactions with no EC category are not included in the calculations. CornCyc unique reactions refer to all reactions that were unique to the CornCyc when compared to MaizeCyc, and vice versa. For MetaCyc and BRENDA, all reactions, including those not found in plants, were included

While 1,857 reactions were found in both CornCyc and MaizeCyc, CornCyc contains 1,245 reactions not present in MaizeCyc, and MaizeCyc contains 417 reactions not present in CornCyc (Fig. 3 e). In order to determine if the differences in reaction content reflect differences in coverage of reaction space, we compared the distribution of Enzyme Commission (EC) categories for the reactions in each resource. Reactions were assigned to EC categories using their top-level EC class. We compared the total reaction content of CornCyc and MaizeCyc to the portion of reactions unique to CornCyc and MaizeCyc, as well as the total reaction content of BRENDA [17] and MetaCyc [23] (Fig. 4). MetaCyc is the source reactions from which CornCyc and MaizeCyc imported their reaction information, while BRENDA contains a comprehensive source of enzyme information derived from literature. MetaCyc, CornCyc, and MaizeCyc frequencies are distributed similarly, whereas frequency distribution for BRENDA is lower in EC 1 and EC 2 and much higher in EC 3.

Table 2 shows that the distribution and overlap of reactions categorized by top-level EC category for CornCyc and MaizeCyc follow a similar trend. Comparing the reactions unique to CornCyc and MaizeCyc reveals that CornCyc has stronger representation than MaizeCyc in each category. A total of 216 unique reactions in CornCyc and 109 in MaizeCyc were not assigned an EC number. Reactions might be missing an EC number in three cases: 1) the reaction is pending review by the EC commission, 2) the reaction is hypothetical without an experimentally characterized enzyme activity, or 3) the reaction is not associated with an enzyme such as the case for some transport reactions. CornCyc has more unique reactions than MaizeCyc in all EC categories.

**Table 2:** Comparison of reaction and EC number statistics for all reactions in CornCyc 4.0 and MaizeCyc 2.2

	Overlap	Unique to CornCyc	Unique to MaizeCyc
Oxidoreductases (EC 1)	496	356	109
Transferases (EC 2)	540	354	101
Hydrolases (EC 3)	282	195	55
Lyases (EC 4)	141	63	28
Isomerases (EC 5)	73	30	5
Ligases (EC 6)	80	33	10
Unclassified (No EC Number)	244	215	127
Total Reactions	1,856	1,246	435

We compared the compounds in both databases for small, non-elemental molecules (i.e., excluding proteins, DNA/RNA, etc.). Since compounds are imported into the CornCyc and MaizeCyc from MetaCyc, we do not expect them to be intrinsically unique in one resource except when the two resources contain reactions catalyzing compounds unique to those reactions. As expected, as CornCyc has more unique reactions, it also contains significantly more small-molecule compounds than MaizeCyc, providing a greater coverage of the compound space (Fig. 3 d). The number of unique reactions has a direct effect on pathway coverage as well: CornCyc and MaizeCyc have 328 pathways in common with 177 and 81 pathways unique to CornCyc and MaizeCyc, respectively (Fig. 3 f).

### The level and quality of manual curation differentiates metabolic databases

Manual curation is a powerful approach for ensuring consistency and accuracy of a database. Unfortunately, the time-consuming and expensive nature of curation means that only limited parts of a data resource will receive manual review. In the case of CornCyc and MaizeCyc, their content was first populated with computationally predicted annotations using their respective annotation pipelines. This content is then reviewed in an ongoing curation effort to integrate literature-supported experimental annotation into the metabolic resources.

The current version of CornCyc (version 7.0) has 114 proteins and 84 pathways with experimental support. One area of MaizeCyc that has received considerable manual curation effort is Gene Ontology (GO) annotations. Because GO annotations are important for researchers interested in gene function, we previously developed a tool to migrate GO annotations between Pathway Tools-based metabolic databases [25]. Previous work reported 789 experimentally verified GO assignments to proteins in MaizeCyc, of which 179 were matched and transferred to CornCyc by using this tool [25].

### CornCyc and MaizeCyc show distinct differences on the pathway level: C4 photosynthesis pathway

**Fig. 5:**
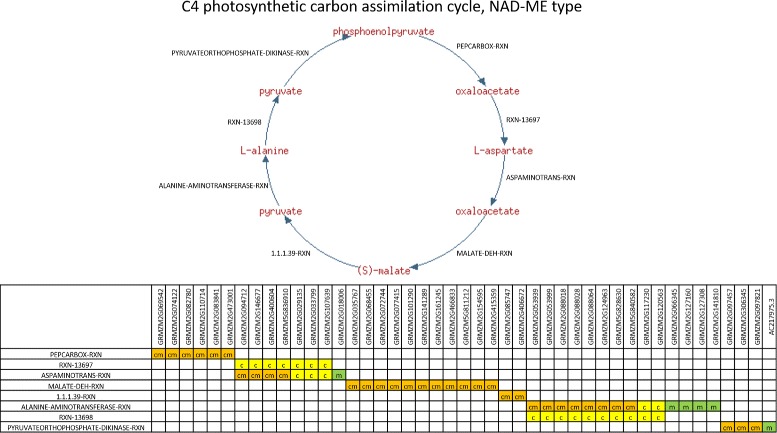
The gene-reaction relationships for reactions in the C4 photosynthesis pathway PWY-7115. While the pathway and reaction-pathway membership (*above*) remain the same for both resources, the gene-reaction relationships differ. Each gene-reaction pair (*below*) indicates if the pairing is found in CornCyc (*yellow*), MaizeCyc (*green*), or both resources (*orange*)

To demonstrate how initial enzymatic functional annotations can lead to differences in a given pathway, we used the C4 photosynthesis pathway (the pathway PWY-7115) as an example, as the pathway is present in both resources. In Fig. 5, the top panel shows the pathway diagram that displays the reaction set. At the bottom, a matrix displays which genes are linked to which reactions in this pathway. A yellow ‘c’ designates a gene which was linked to the given reaction in CornCyc, while a green ‘m’ designates a gene which was linked to the given reaction in MaizeCyc. An orange ‘cm’ means that CornCyc and MaizeCyc agree on a gene-reaction pairing. This example visually illustrates how different gene-reaction pairs in the C4 photosynthesis pathway for both resources. In this example, CornCyc predicts enzymes for the reactions RXN-13697 (catalyzed by aspartate transaminase) and RXN-13698 (catalyzed by alanine transaminase) while MaizeCyc does not predict any enzymes for these reactions.

## Conclusions

The availability of genome-wide metabolic pathway resources provides a systems-level view of the chemical interactions in a cell, which creates phenotypes of interest. When a metabolic pathway resource is developed and made publicly available, scientists can then construct a network of interactions around their enzymes of interest, and build further hypotheses based on the annotations assigned to the genes and proteins. For example, when an enzyme of interest is discovered to be differentially expressed and hypothesized to play a critical role in cellular processes, the next step is often to gather its functional annotations from several database resources for further analyses. Therefore, it is highly desirable for a metabolic pathway resource to have annotations for larger numbers of enzymes. A higher coverage of the genome-wide enzyme space, however, does not automatically translate into a higher accuracy of prediction for those annotations. Most of these annotations are generated through computational pipelines that involve multiple processing steps, and each step can contribute the final quality of a metabolic pathway resource. A larger number of functional assignments can indeed provide a higher number of correct assignments (i.e., true positives), but it can also introduce a higher number of wrong assignments (i.e., false positives).

CornCyc 4.0 and MaizeCyc 2.2 are based on the same maize genome assembly version (B73 RefGen_v2), and reaction and pathway mapping were done using the Pathway Tools software suite that heavily uses an “encyclopedia” of pathways “from all domains of life” called MetaCyc [23]. CornCyc and MaizeCyc, however, were created by two different research groups based on their pipeline for enzymatic function assignments. In this work, we harnessed the availability of these two distinct metabolic pathway resources for maize in order to compare how initial enzymatic function assignments influence the final products that the biologists commonly use in their research.

Our results demonstrate that even though both CornCyc and MaizeCyc were constructed using the same gene model set and the same pathway assignment software, they have significantly different content. When we compared both databases in detail, we observed that MaizeCyc contains a larger number of GO annotated genes whereas CornCyc covers a larger metabolic space having more compounds, reactions, and pathways.

We also extracted experimentally determined enzymatic function assignments from UniProt and analyzed how well these assignments were discovered by the computational pipelines used during the development of the resources. We defined performance measures such as precision and recall, and consolidated these results into a single F-measure. F-measure comparison demonstrates that though CornCyc coverage is more limited than that of MaizeCyc in terms of GO-annotated genes, its functional annotations are more stringent, and therefore more reliable for creating further hypotheses. Alternatively, a dataset composed of the merged annotations from both CornCyc and MaizeCyc demonstrates that there is potential benefit to a merged resource which, while less accurate overall than CornCyc, would provide greater coverage than CornCyc or MaizeCyc at a higher accuracy than MaizeCyc alone.

To conclude, computational pipelines used in the initial enzymatic function assignments can have a large impact on the scope and quality of the metabolic pathway resources. The features of these pipelines determine the final accuracy and quality of these resources. Given the large divergence between CornCyc and MaizeCyc after starting from the same gene set, users of other metabolic resources should give additional scrutiny to the methods used in the generation of the resource.

## Additional file

Additional file 1 Method for extracting and comparing data from CornCyc and MaizeCyc. This document describes how CornCyc and MaizeCyc data were selected, extracted, and filtered and/or modified before comparison. The five major data types, Genes, Proteins, Compounds, Pathways, and Reactions were each handled in unique ways. (PDF 342 kb)

## Abbreviations

API: Application programming interface
E2P2: Ensemble enzyme prediction pipeline
EC: Enzyme commission
FGS: Filtered gene set
FN: False negative
FP: False positive
GO: Gene ontology
PMN: Plant metabolic network
RPSD: Reference protein sequence dataset
SAVI: Semi-Automated validation and integration
TN: True negative
TP: True positive
