# Supplementary material for: The quality of metabolic pathway resources depends on initial enzymatic function assignments: a case for maize
Source: BMC Syst Biol. 2016 Nov 29;10:129. doi: 10.1186/s12918-016-0369-x (PMC5129634; doi:10.1186/s12918-016-0369-x)
Supplement: Additional file 1 — Method for extracting and comparing data from CornCyc and MaizeCyc. This document describes how CornCyc and MaizeCyc data were selected, extracted, and filtered and/or modified before comparison. The five major data types, Genes, Proteins, Compounds, Pathways, and Reactions were each handled in unique ways. (PDF 342 kb) [file 12918_2016_369_MOESM1_ESM.pdf]

The Quality of Metabolic Pathway Resources Depends on Initial Enzymatic Function Assignments: A Case for Maize

Jesse R. Walsh[1,2], Mary L. Schaeffer[3], Peifen Zhang[4], Seung Y. Rhee[4], Julie A. Dickerson[1,2], Taner Z. Sen[1,5,6*]
[1] Bioinformatics and Computational Biology Program, Iowa State University, Ames, IA, USA
[2] Electrical and Computer Engineering Department, Iowa State University, Ames, IA, USA
[3] U.S. Department of Agriculture, Agricultural Research Service, Plant Genetics Research Unit and Division of Plant Sciences, University of Missouri, Columbia, MO
[4] Department of Plant Biology, Carnegie Institution for Science, Stanford, CA USA
[5] U.S. Department of Agriculture, Agricultural Research Service, Corn Insects and Crop Genetics Research Unit, Iowa State University, Ames, IA, USA
[6] Department of Genetics, Development and Cell Biology, Iowa State University, Ames, IA, USA

Supplemental Materials

Database Schema Structure

A Pathway Tools-based BioCyc database is organized as a collection of frames. A frame stores information representing either a single biological entity such as a metabolite or a gene, or a biological interaction such as a regulatory event or a pathway. Frames have named properties called slots, which describe the object they represent, such as the name of a gene, the molecular structure of a metabolite, or the reactions in a pathway. Frames are organized within the BioCyc database using the Pathway Tools ontology. The root of the Pathway Tools ontology is the frame "Things". The frame "Things" has only one child, the frame "Frames." Below "Frames" the ontology branches into major divisions, including biological entities such as "Chemicals" and "Enzymatic-Reactions", as well as metadata and annotation data such as "Databases", "People", and "Publications." The ontology is structured such that every frame (except the root frame "Things") can have one or more parent frames and zero or more child frames.

Within the frame ontology, a frame can represent either a class of frames or an instance frame. Instance frames contain specific information about biological objects, such as a particular gene or a specific chemical compound. Class frames group similar instances together and describe the general properties of the group. Class frames such as "All Genes" or "Pathways" define the properties that genes or pathways should specify. The class frames serve multiple purposes, including organizing the data within the resource so that it can be more easily found and referenced by both Pathway Tools and users, providing internal documentation describing what objects are represented by that class, and serving as a template for creating new frames of that class type.

Data Structure Comparison

Table 1. The distribution of classes in CornCyc v4.0 and MaizeCyc v2.2 after propagating MetaCyc updates from Pathway Tools v17.5. Although the Pathway Tools ontologies for CornCyc and MaizeCyc are very similar, there are slight differences in protein, compound, and pathway classes between the two resources.

	CornCyc4.0 Only	Overlap	MaizeCyc2.2 Only
Gene Classes	0	247	0
Proteins Classes	15	400	6
Compound Classes	9	2,844	5
Reaction Classes	0	39	0
Pathway Classes	0	530	4

Classes represent the core database structure of all BioCyc databases. They are created automatically during the database generation process by Pathologic as part of the Pathway Tools software, which selectively imports them from MetaCyc database based on enzymatic function assignments. Since classes are imported on an as-needed basis, the inclusion or exclusion of certain class frames can be an indication of differences in genomic and metabolic representation between two databases. If a class frame appears in two databases generated with the same Pathway Tools version, the content of the class frame is not expected to differ. The class frame information is updated only when MetaCyc is updated. Table 1 shows where the class structure differs between CornCyc and MaizeCyc.

Gene and Protein Comparison

The Pathway Tools ontology defines several types of genes, but for our purposes we ignore the Phantom-Genes and Pseudo-Genes categories in the schema and consider only genes classified under the Genes class. The red boxes in Figure 1 represent Pathway Tools ontology class frames. Class frames in CornCyc and MaizeCyc have very similar content. Figure 1 shows the ontology structure starting at Genes. The red boxes (class frames) are identical in both CornCyc and MaizeCyc, showing the similarity in their organization. However, the blue boxes (instance frames) are not only labeled differently, they are placed in different locations in the Pathway Tools ontology. CornCyc, for example, has several genes placed directly under the Genes class, while MaizeCyc has a larger number of genes placed under the Unclassified-Genes class. There are 214 unclassified genes in MaizeCyc, but only 4 in CornCyc, mainly due to differences in computational pipelines and, to a smaller extent, in manual curation.

MaizeCyc and CornCyc classify their data differently within the Pathway Tools Ontology. MaizeCyc includes all gene data under the "ORF" category, while CornCyc includes most of its genes under the "ORF" category and some under the "Genes" category (see Figure 1). This suggests that the most appropriate ontology category to compare gene data is at the "Genes" class and below. In CornCyc, 43 genes are stored directly under the "Genes" category.

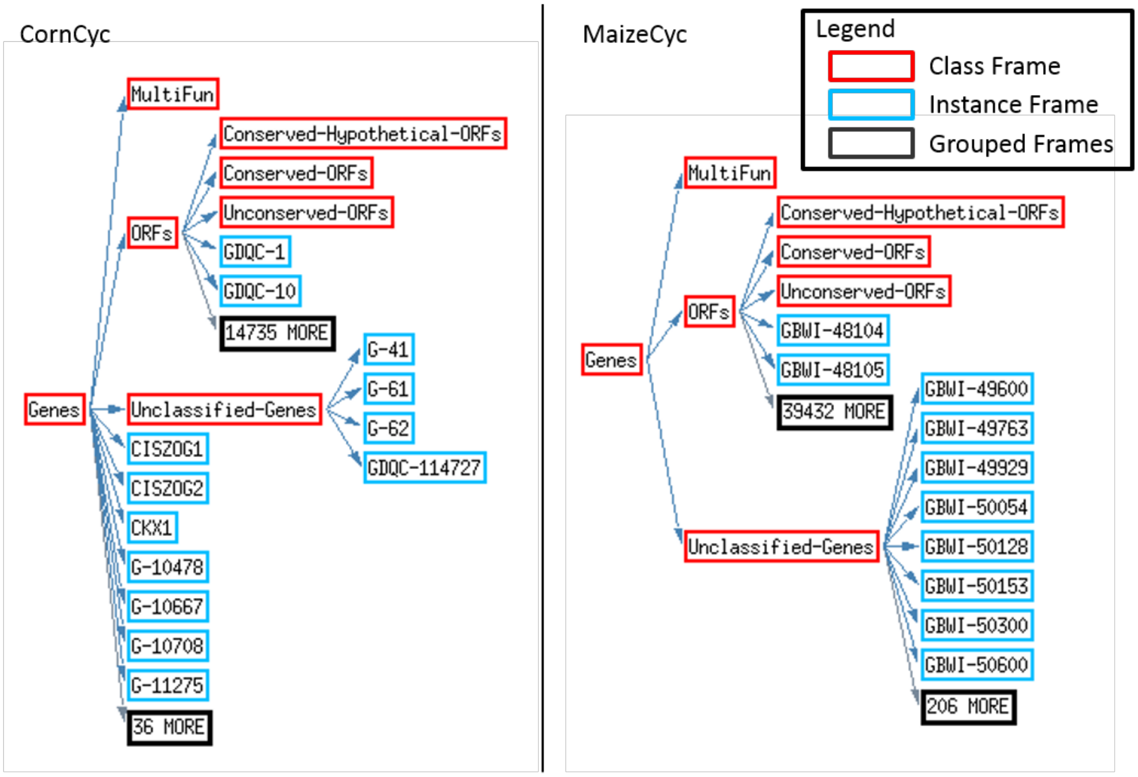

Figure S1. An example of the Pathway Tools Ontology for (Left) CornCyc 4.0 and (Right) MaizeCyc 2.2. Red class frames represent the Pathway Tools Ontology structure, while blue instance frames represent information about Maize genes. The text represents the internal identifier for the genes. In addition to differences in information content between these two resources, the gene information is sometimes stored in different locations despite having the same basic Pathway Tools Ontology structure. The structure categorizes data content. MaizeCyc lists more genes as uncategorized. CornCyc includes 43 genes that are directly under the Genes category instead of the ORFs category.

The text in the boxes of Figure 1 represents internal identifiers for the frames in CornCyc and MaizeCyc. CornCyc internally assigned GDQC prefixes for genes, while MaizeCyc assigned GBWI prefixes for the same genes. Therefore, we are unable to match genes between CornCyc and MaizeCyc using frame id. Since the genes in both databases were annotated with their gene model names and transcript number suffixes, we are able to use a modified synonym search to match genes. For each gene, the gene model name was identified and the transcript-specific suffix stripped from the name. The resulting gene model names were matched between CornCyc and MaizeCyc.

CornCyc represents multiple splice variants for genes, while MaizeCyc only stores a single canonical transcript per gene for 99.5% of the genes it contains. Since Pathway Tools does not specify a standard format for storing transcript data, the transcript information is stored in gene objects using gene names suffixed with either a "_P##" (for protein) or "_T##" (for transcript), where ## represents a two-digit number to identify a given transcript. When matching transcripts, we homogenized the names by ensuring that all transcripts used the "T" suffix instead of the "P" suffix.

Compound, Reaction, and Pathway Comparison

Common names of reactions are not consistent between the public versions of both resources. This can be due to either typographical differences or missing information either resource. Approximately 63

reactions were not given a descriptive common name in CornCyc but were given one in MaizeCyc, and 66 reactions were not given a descriptive name in MaizeCyc but were given one in CornCyc. Examples of typographical differences include the use of special characters (beta-carotene 3-hydroxylase vs. β-carotene 3-hydroxylase), formatting markup (tryptophan\textless em\textgreater N\textless /em\textgreater -monooxygenase vs. tryptophan N-monooxygenase), and equivalent names (2-oxo-3-phenylpropanoate dioxygenase vs. phenylpyruvate dioxygenase).

Compounds are imported from MetaCyc during the early database creation steps of Pathologic on an as-needed basis. Since both CornCyc and MaizeCyc imported compounds from MetaCyc, we were able to match them based on frame ID's. For each compound matched in this way, we checked to see if the compound names and InChI strings matched. InChI strings are designed to facilitate computational representations of chemical compounds, therefore InChI matches should verify that the compounds have the same structure for more accurate matching. We found that many compounds which matched based on frame ID did not match name or InChI string.

Pathways are imported from MetaCyc during the Pathologic inference steps based on the reaction complement of the database. Since both CornCyc and MaizeCyc imported pathways from MetaCyc, we were able to match pathways based on frame IDs. Superpathways were excluded from the matching step as they simply represent a collection of standard pathways. The process of upgrading the MetaCyc content from each database effectively removes the inconsistencies in compounds, reactions, and pathways.

Protein-EC number Overlap

As a result of their independent development pipelines, it is not surprising that conflicting data was frequently observed between CornCyc and MaizeCyc. Aside from manual curation, examples of conflicting information included differences in chemical InChI codes, differences in GO Term annotations, renamed pathways, and differences in the gene and reaction membership for certain pathways. Many of these inconsistencies were related to the version of MetaCyc used during the initial creation of CornCyc and MaizeCyc, and can be brought back into alignment through the MetaCyc update and pathway rescoring procedures.

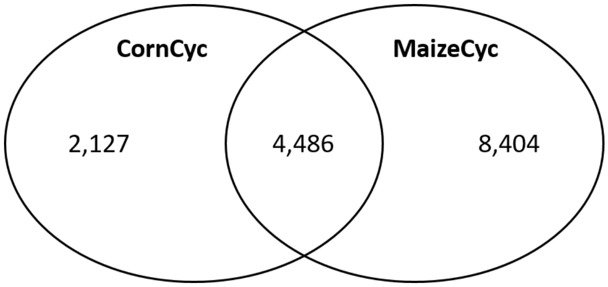

Figure S2. The overlap in protein-EC annotations between CornCyc and MaizeCyc. For the 5,425 proteins common to both CornCyc and MaizeCyc, the distribution in protein-EC assignments is shown.

An example of conflicting information not removed by the update and rescoring process is protein-EC annotations. Figure 2 shows conflicting EC annotations between two resources for the 5,425 common proteins. For the proteins that are matched between CornCyc and MaizeCyc, we counted the number of

times EC assignments for these proteins on a protein by protein basis agree or disagree. We found 4,486 instances where a protein-EC annotation appeared in both databases. There were 2,127 instances where an EC number was assigned to a protein in CornCyc but not assigned to the same protein in MaizeCyc. Conversely, there were 8,404 instances unique in MaizeCyc. We observed that there are more EC annotations on average per protein in MaizeCyc (2.38 EC annotations per protein) than in CornCyc (1.22 EC annotations per protein). The differences in the reaction and pathway assignments in CornCyc and MaizeCyc are primarily due to the protein annotation pipelines.
